# The Rostral Ventrolateral Medulla Input to Central Amygdala Regulates Anxiety‐Like Behaviors in Mice for Chronic Light Exposure

**DOI:** 10.1002/cns.70775

**Published:** 2026-02-05

**Authors:** Jia‐Ni Jing, Xing Tan, Chou Xu, Chao Yuan, Hao Fan, Wei‐Zhong Wang

**Affiliations:** ^1^ Center of Cognition and Brain Science Beijing Institute of Basic Medical Sciences Beijing China; ^2^ Naval Medical Center Naval Medical University (Second Military Medical University) Shanghai China; ^3^ Department of Critical Care Medicine The 983rd Hospital of the Joint Logistics Support Force of PLA Tianjin China

**Keywords:** anxiety‐like behaviors, blood pressure, central amygdala, chronic light exposure, rostral ventrolateral medulla

## Abstract

**Background:**

Emerging evidence increasingly links environmental light exposure to the development of anxiety disorder. The rostral ventrolateral medulla (RVLM), a key brainstem center that regulates sympathetic outflow and blood pressure (BP), has been implicated in the autonomic dysregulation frequently observed in anxiety. High blood pressure is a recognized exacerbating factor for anxiety‐related pathology. Although aberrant projections from the central amygdala (CeA) to brainstem regions have been reported in multiple mood disorders, it remains unclear whether the CeA receives input from the RVLM and to what extent the RVLM's excitation influences anxiety symptoms induced by chronic light exposure (CL).

**Methods:**

We employed a chronic mild light (250–300 lx) for 4 weeks to induce anxiety‐like behaviors in mice. Using a tail‐cuff system and ELISA assay, we assessed CL mice's BP and plasma catecholamine levels. Immunofluorescence was utilized to unravel the neuronal c‐fos expression in the RVLM and CeA. Combining retrograde virus tracing technology, chemogenetic and optogenetic manipulations in freely moving mice to examine the effects of regulating RVLM‐CeA pathway on anxiety‐like behaviors induced by chronic light.

**Results:**

Chronic light exposure in mice induces elevated blood pressure and elevated neuronal activity, which are more pronounced in the RVLM than in the CeA. Chemogenetic inhibition of RVLM neurons markedly attenuated CL‐induced anxiety‐like behaviors. Moreover, optogenetic inhibition of the RVLM→CeA pathway reduced anxiety phenotypes in CL‐exposed mice, whereas optogenetic activation of this pathway in normal mice acutely triggered anxiety‐like behaviors.

**Conclusions:**

These findings demonstrate that the enhanced excitatory signaling within the RVLM→CeA circuit underlies the development of anxiety‐like behaviors induced by chronic light, revealing a novel mechanism by which the RVLM regulates light‐related affective dysfunction.

## Graphical abstract

Jing et al. demonstrate that the CeA receives input from the RVLM in normal mice, and the chronic light exposure induced more pronounced elevated neuronal activity in the RVLM than the CeA. The enhanced excitatory input from the RVLM to CeA induced anxiety‐like behaviors in mice, revealing a novel circuit by which the RVLM regulates light‐related anxiety dysfunction.

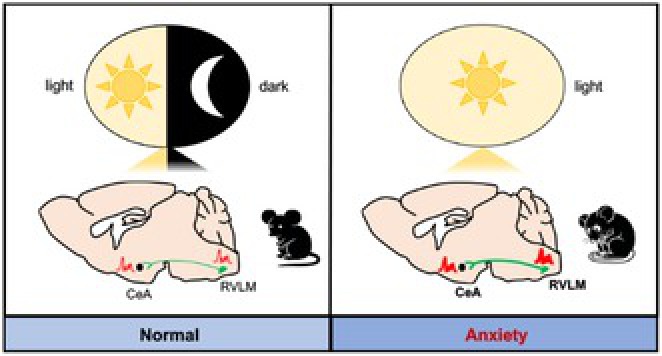

## 1 Introduction

It has been documented that anxiety disorders are a prevalent group of psychiatric conditions characterized by pathological fear, hyperarousal, and avoidance behaviors. With a global lifetime prevalence exceeding 30% [1], they impose a profound multisystem disease burden. Physiologically, sustained activation of the hypothalamic–pituitary–adrenal (HPA) axis promotes glucocorticoid resistance and systemic inflammation, thereby increasing the risk of cardiovascular disease, including hypertension, myocardial infarction, and metabolic syndrome [2]. Despite major advances in pharmacotherapy and psychotherapy, the precise neurobiological mechanisms underlying anxiety disorders remain incompletely understood, particularly those associated with environmental risk factors such as light pollution.

In modern societies, excessive exposure to artificial light at night (ALAN) disrupts endogenous circadian rhythms and has been linked to an increasing incidence of mood disorders in both epidemiological and preclinical studies [3, 4, 5, 6]. Prolonged light exposure alters neuronal activity in brain regions involved in emotional regulation, including the prefrontal cortex, hippocampus, hypothalamus, and brainstem [4]. Environmental light exposure, particularly artificial illumination during the night, may therefore represent a critical yet underappreciated contributor to anxious pathophysiology [7, 8, 9, 10]. However, the specific neural circuits through which chronic light exposure promotes anxiety‐like symptoms remain poorly delineated.

The sympathetic nervous system and blood pressure (BP) are frequently elevated in mood disorders, including anxiety and depression [11, 12, 13]. Anxiety and depression are recognized risk factors for the development of hypertension, which comorbidity rates ranging from 15% to 50% [14]. Moreover, the prevalence of depression is significantly higher among patients with hypertension compared to those with normal BP [15]. Importantly, experimental studies suggest that hypertension is not merely a comorbidity but may be mechanistically linked to anxiety via shared neural circuitry involving the rostral ventrolateral medulla (RVLM) [16, 17, 18], a key brainstem region responsible for maintaining tonic sympathetic outflow and basal BP [19, 20]. Chronic stressors, particularly sustained light exposure, can elicit prolonged sympathetic excitation and may reflect increased neuronal activity with in RVLM, resulting in elevated BP and heart rate [21]. Given the dual role of the RVLM in autonomic regulation and blood pressure, it represents a compelling candidate for mediating the emotional consequences of chronic light exposure.

Anatomical and functional studies have revealed that the RVLM receives inputs from and sends projections to several limbic structures, including the central amygdala (CeA), a core component of the mesolimbic system involved in emotional regulation, especially fear and anxiety [22, 23, 24, 25]. The CeA also modulates cardiovascular responses under stress, supporting the existence of a functional RVLM→CeA interaction [16, 26]. However, whether this pathway contributes to the pathogenesis of anxiety‐like behaviors under chronic light‐induced stress remains largely unexplored. Based on this background, we hypothesize that chronic light exposure abnormally activates the RVLM→CeA circuit, thereby facilitating the development of anxiety‐like behaviors. To test this hypothesis, we combined viral tracing and immunofluorescence to map RVLM to CeA projections, and used chemogenetic and optogenetic techniques to manipulate this pathway. These experiments aimed to determine whether the RVLM→CeA circuit functions as a key neural substrate through which environmental light exposure modulates mood regulation.

## 2 Materials and Methods

### 2.1 Chronic Light Exposure Procedure

Light, as an independent factor, directly influences emotional behaviors irrespective of circadian rhythms [3, 27]. In industrial or occupational environments, mild light intensities are physiologically necessary and harmless to humans in daily life [28, 29]. However, some night‐shift workers, special forces soldiers, and polar expedition personnel often expose themselves to prolonged nighttime illumination [30], causing severe mood disorders. In rodents, constant, mild light intensities do not alter visual sensitivity but contribute to negative emotional states [31, 32]. Therefore, we employed chronic mild light intensities of 250–300 Lux to induce negative emotional behaviors in mice. Two distinct lighting conditions were employed in this study as follows: control or chronic light exposure (CL) mice for 4 weeks. For control mice, housing conditions included a controlled 12‐h light/12‐h dark cycle, with lights on at 07:00, administered by a programmable electronic timer. The CL mice were raised in 24 h light (250~300 Lux white ambient illumination). To avoid artifacts arising from the light delivery system itself, a neutral lighting regimen with minimized spectral and intensity biases was employed. Locomotor activities were assessed using a customized wheel‐running system (ACTIMETRICS INSTRUMENTS). Male C57BL/6J mice aged 6–8 weeks were used in this study. The animals were obtained from Beijing Sibeifu Biotechnology Co. Ltd. Mice were housed in groups of five per cage under standard conditions (ambient temperature at 23°C–25°C) with ad libitum access to standard laboratory chow and water. All animal experiments complied with the NIH Guide for the Care and Use of Laboratory Animals and were approved by the Institutional Animal Care and Use Committee (IACUC) of the Military Medical Research Institute (approval ID: IACUC‐JSRZ‐23‐0201).

### 2.2 Stereotaxic Surgery, Virus Injection and Optical Fiber Implantation

Mice were intraperitoneally anesthetized with 0.7% pentobarbital sodium injection (injection dose: 0.1 mL/10 g) and were mounted in a stereotaxic frame (68,018, RWD). For viral delivery, recombinant AAVs were stereotaxically infused into the RVLM and CeA using a microprocessor‐controlled injection system (LEGATO 130, kd Scientific) at volumes ranging from 100 to 400 nL with an infusion rate of 40 nL/s. The injection needle was retained in situ for an additional 5–10 min to minimize backflow and facilitate adequate diffusion of the viral particles. For in vivo optogenetic stimulation, an optical fiber (200 μm diameter, 0.37 numerical aperture; RWD) was implanted above the RVLM using stereotaxic guidance following a minimum 3‐week post‐injection recovery period. Under isoflurane anesthesia, the chronically implanted optical fiber (Shanghai Fib‐laser) was secured to the skull using a dental cement cap that incorporated the fiber optic cannula and its protective sleeve. Following this, a post‐procedural recovery period of at least 30 min was provided in the home cage. An optogenetic system stimulator (IOS‐473/IOS‐589 RWD) (blue light: 473 nm; yellow light: 589 nm; 5 mW; 15‐ms pulse duration; 20 Hz) was delivered to modulate RVLM‐derived inputs to the CeA. The same optical delivery parameters were applied to control mice. The following coordinates were used: RVLM (6.60 mm anterior to bregma, 1.30 mm lateral to the midline, 5.55 mm below the pia), CeA (1.07 mm posterior to bregma, 2.75 mm lateral to the midline, 4.6 ~ 4.8 mm below the pia). Stereotaxic coordinates were appropriately calibrated according to the developmental stage and individual neuroanatomical variations of each mouse. The following recombinant adeno‐associated viral (AAV) vectors were employed in this procedure from the BrainVTA company: AAVretro‐hSyn‐EGFP‐3XFlag‐WPRE‐hGH pA (4.2 × 10^12^ vector genomes/mL), AAVretro‐DBH‐EYFP‐WPRE‐hGH polyA (6.84 × 10^12^ vector genomes/mL), AAVretro‐hSyn‐NLS‐CRE‐WPRE‐SV40 polyA (2.37 × 10^13^ vector genomes/mL), rAAV‐hSyn‐DIO‐ChrimsonR‐mCherry‐WPREs (2.48 × 10^12^ vector genomes/mL), rAAV‐hSyn‐DIO‐eNpHR3.0‐EGFP‐WPREs (2.48 × 10^12^ vector genomes/mL).

### 2.3 Chemogenetic Manipulations

For chemogenetic experimentation, mice were subjected to a 3‐day handling acclimation period before behavioral testing to minimize stress associated with human interaction. Viral vectors (recombinant AAVs) were stereotaxically delivered into target brain regions using the methodology described above. On the day of testing, mice were transferred to the behavioral testing room and allowed to habituate to the novel environment for a period of 4 h prior to experimentation. For chemogenetic manipulation via systemic ligand administration, mice were administered clozapine‐N‐oxide (CNO) intraperitoneally at a dose of 2 mg/kg (BrainVTA) under isoflurane anesthesia 30 min prior to behavioral assessment. Following behavioral tests, mice were euthanized for verification of viral injection sites. Animals exhibiting inaccurate viral targeting were omitted from further statistical processing. The following AAV viruses were used from the BrainVTA company in this step: rAAV‐TH‐mcherry‐WPREs (2.3 × 10^12^ vector genomes/mL), rAAV‐hSyn‐mcherry‐WPRE‐hGH pA (2.3 × 10^12^ vector genomes/mL), rAAV‐hSyn‐hM4D(Gi)‐mcherry‐WPREs (5.85 × 10^12^ vector genomes/mL), rAAV‐TH‐hM4D(Gi)‐mcherry‐WPREs (2.3 × 1012 vector genomes/mL).

### 2.4 Tissue Processing, Immunofluorescence and Imaging

Mice were deeply anesthetized with sodium pentobarbital (0.7%) and perfused with 4% paraformaldehyde (PFA) in 0.1 M phosphate‐buffered saline (PBS). For immunohistochemical analysis, brains were carefully removed and post‐fixed in 4% paraformaldehyde (PFA) at 4°C for 24 h. After thorough washing in phosphate‐buffered saline (PBS), 30–40 μm coronal sections were prepared using a cryostat microtome (CM1950, Leica, Germany). Every third section was selected and permeabilized with 0.2% Triton X‐100 in PBS for 1 h at room temperature (RT), followed by blocking in a solution containing 0.05% Triton X‐100 and 10% bovine serum albumin (BSA) in PBS for 1.5 h at RT. After additional PBS washes, sections were incubated overnight at 4°C with primary antibodies—rabbit anti‐c‐Fos (1:1000; Synaptic Systems) and mouse anti‐Cre (1:1000; Millipore)—diluted in PBS containing 0.3% Triton X‐100. Following three washes in PBS, sections were incubated for 1.5 h at RT with fluorescent secondary antibodies (goat anti‐rabbit 488/546 and goat anti‐mouse 546; 1:2000; Life Technologies). Finally, sections were rinsed, mounted on glass slides, air‐dried, and cover slipped using glycerol: TBS (3: 1) containing Hoechst 33342 (1:1000; Thermo Fisher Scientific) for nuclear counterstaining. Imaging was performed using a Nikon A1 laser scanning confocal microscope (Tokyo, Japan) with 20× and 60× objectives.

### 2.5 Open Field Test (OFT)

A mouse spontaneous activity monitoring system (VisuTrac, Shanghai) is used to monitor the activity characteristics of mice in their natural state. The open field test (OFT) was conducted in a transparent acrylic arena (50 × 50 × 20 cm), and behavioral performance was automatically recorded and analyzed using the DigBehv video tracking system (Jiliang, China). Each mouse was placed in the center of the arena at the beginning of the trial and allowed to explore freely for 30 min. After the mice become familiar with the environment, the duration in the central zone and total ambulatory distance traveled within 10 min are recorded and analyzed. The central area (25 cm × 25 cm) is defined as a square of 50% of the total area of OFT. The CL mice present a reduced total distance and have less time in the central than the control mice.

### 2.6 Elevated Plus Maze (EPM)

The EPM was comprised of a central platform (5 × 5 cm), two enclosed arms (35 × 5 × 15 cm), and two open arms (35 × 5 cm) positioned 50 cm above the floor. Each mouse was positioned in the central platform facing an open arm and granted 5 min of unrestricted exploration. Behavioral sessions were recorded using a video tracking system (Shanghai xinruan, China) for subsequent analysis. Motor activity and movement trajectories were quantified offline. The time spent in the open arms and the total number of arm entries were calculated. The maze was thoroughly cleaned between trials with 75% ethanol solution. Trajectory heatmaps were generated to visualize locomotor patterns. Compared with control mice, CL mice displayed significantly reduced total locomotor activity and diminished open‐arm exploration time in the EPM.

### 2.7 Sucrose Preference Test (SPT)

The decreased sucrose consumption is thought to reflect anhedonia symptoms. Before the experiment began, the animals were trained to feed from two bottles of water for 36 h, then one bottle of 1% (w/v) sugar solution and one bottle of ordinary water, switching the bottles every 12 h. After the training, the mice were restricted from drinking water for 12 h, then fed a bottle of 1% sugar water and a bottle of regular water for 24 h, still switching the bottles every 12 h. Finally, the sucrose preference is calculated by combining the total consumption of sugar/the total consumption of water and sucrose. There is a decreased sucrose intake (less than 60% in sucrose consumption) in the CL mice compared with the control mice, which exhibited anhedonia behavior.

### 2.8 Measurements of BP, HR and Plasma Hormone Examination

Non‐invasive BP was assessed in conscious mice using a tail‐cuff system (BP‐2010AUL; Beijing Biotech for R&D) to record systolic blood pressure (SBP), diastolic blood pressure (DBP), and mean arterial pressure (MAP), as well as heart rate (HR). Prior to measurement, mice were acclimated to the restraint for 15 min. Reported values represent the average of seven consecutive measurements. BP and HR were measured daily during the final week following chronic CL exposure. Nocturnal blood samples were collected. Plasma glucocorticoid, melatonin, corticosterone, and catecholamine substances (norepinephrine and epinephrine) were tested with an ELISA kit (Jiangsu ELISA Industrial Co. Ltd.). Experiments were conducted carefully following the instructions from merchandise.

### 2.9 Quantification and Statistical Analysis

No formal power analysis was used to predetermine sample sizes; instead, cohort sizes were chosen based on established protocols from previous studies in the field. All behavioral data were analyzed using GraphPad Prism (v9.0). Comparisons between two groups exhibiting homoscedasticity were performed using an unpaired Student's *t*‐test. For comparisons among three or more groups, one‐way analysis of variance (ANOVA) was employed, followed by Tukey's honestly significant difference (HSD) post hoc test. A *p*‐value of less than 0.05 was considered statistically significant (*p < 0.05).

## 3 Results

### 3.1 Mice in CL Emerged Anxiety Symptoms and Elevated Blood Pressure

**FIGURE 1:**
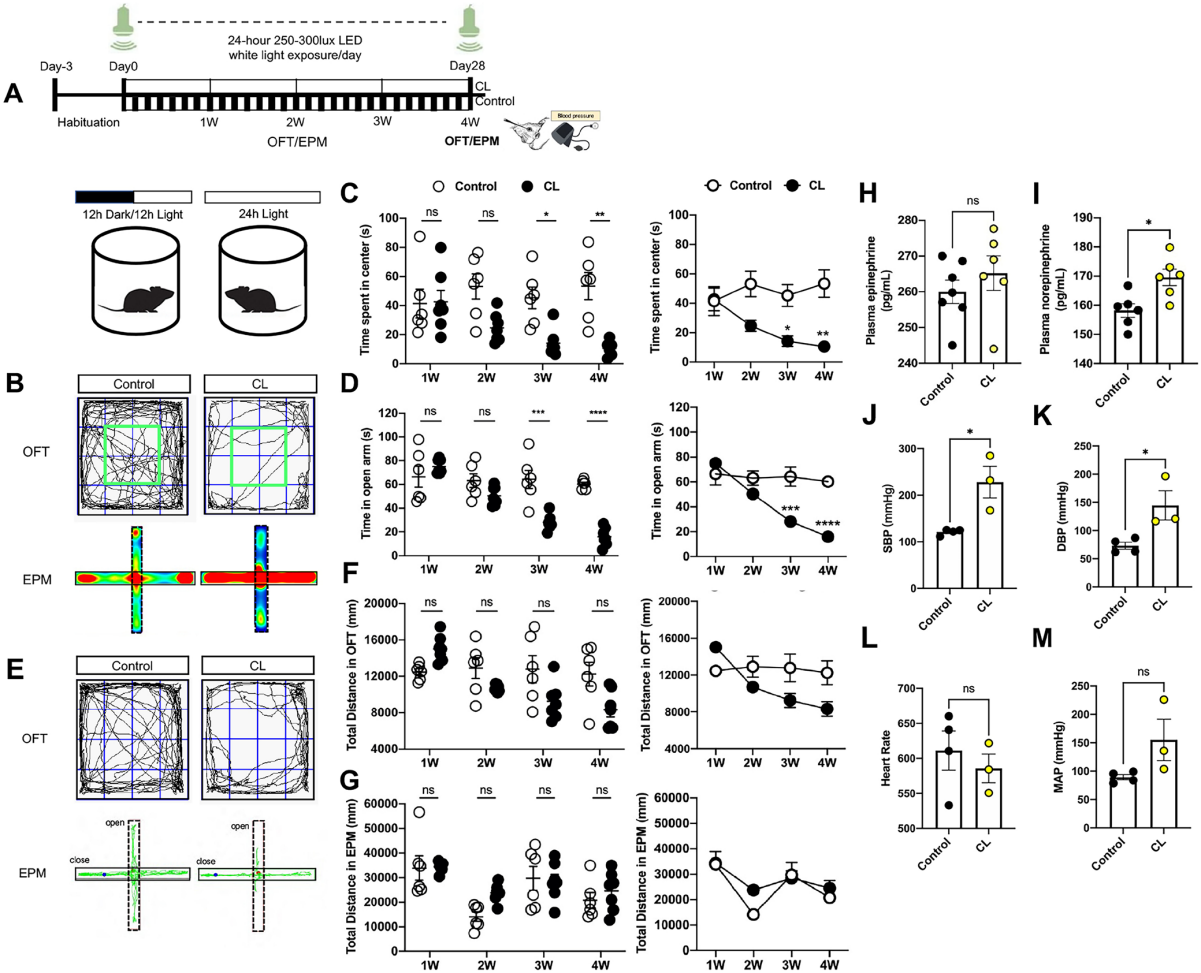
Mice were induced anxiety‐like behaviors and high blood pressure under chronic light exposure (CL). (A) Top: Schedule of light exposure, behavioral test, catecholamines assay and blood pressure measurement; Bottom: Diagram illustrating light exposure in a chamber with controlled lighting. (B) Top: The center movement trajectories during the open field test (OFT); Bottom: The corresponding heatmaps from the elevated plus maze (EPM). (C) Data summarizing the center zone duration of the OFT at various stages of light exposure. (D) Data summarizing the open arm retention time of the EPM during different light exposure phases. (E) Representative movement paths in the OFT (top) and EPM (bottom). (F) Data summarizing cumulative distance traveled in the OFT at different light exposure times. (G) Data summarizing total travel distance in the EPM at different light exposure intervals. (H, I) Plasma concentrations of epinephrine and norepinephrine in mice exposed to constant light compared to Control mice. (J) SBP measurement in the Control and CL mice. (K) DBP measurement in the Control and CL mice. (L) Heart rate measurement in the Control and CL mice. (M) MAP measurement in the Control and CL mice. Data are presented as mean ± SEM. Statistical significance is compared between CL and Control groups: *p < 0.05; **p < 0.01; ***p < 0.001; ****p < 0.0001 in the 3 W, 4 W respectively. Statistical analysis was performed using repeated measurement ANOVA for panels (C), (D), (F), and (G). For these, *n* = 6 Control mice and *n* = 7 CL mice. Analyses corresponding to panels (H, I) were conducted using unpaired *t*‐test (*n* = 6 both in Control and CL mice). For panels (J–M), unpaired *t*‐test was applied (*n* = 4 for Control mice, *n* = 3 for CL mice).

To explore the effects of CL on anxiety disorder and BP, mice were exposed to light daily in a constant light chamber (Figure 1A). First, we employed two well‐validated behavioral paradigms OFT and EPM to diagnose anxiety‐like phenotypes and utilized tail‐cuff system to measure mice's BP. The results have discovered that CL mice exhibited significant decrease in the duration of center zone exploration in the OFT and open arm occupancy in the EPM compared with Control mice (Figure 1B–E). Moreover, the chronic light exposure elicited anxiety‐like behaviors in mice that developed in a time‐dependent manner, emerging during the third week and persisting stably throughout the remainder of the experimental period (Figure 1C,D). In contrast, no statistically significant differences in total travel distance, as measured by cumulative distance traveled (OFT and EPM), were detected between the CL and Control mice throughout the behavioral tests (Figure 1F,G). Further, we have also found that the 24 h sucrose consumption gradually decreased in CL mice at different time points (from 1 week to 4 week), suggesting that chronic light exposure induced anhedonia behavior (Figure S1). After 4 weeks' light exposure, the spontaneous activity of CL mice was significantly decreased in wheel running test (for 24 h) (Figure S2A–D). Second, the plasma glucocorticoid, melatonin and corticosterone levels were examined by ELISA assay. There was significant increase of plasma glucocorticoid and corticosterone in CL mice relative to the Control mice (Figure S2F,G), but there was no obvious difference of plasma melatonin level between the CL and Control mice (Figure S2E), suggesting that chronic light exposure heightens the stress susceptibility of CL mice. Importantly, the current data have showed that the SBP approximately increased by 107 mmHg in CL mice compared with the Control mice and the DBP and MAP also increased (Figure 1J,K,M). Besides, the CL mice presented increased norepinephrine and epinephrine levels compared with the Control mice after 4 weeks' light exposure (Figure 1H,I). In summary, mice in chronic light emerged anxiety symptoms, enhanced BP and increased stress susceptibility. Based on the observed efficacy of a 4‐week light exposure protocol, the chronic light paradigm was adopted for all subsequent experimental procedures, unless otherwise stated.

### 3.2 Chronic Light Exposure Markedly Increased Neuronal Excitation in the CeA and RVLM Regions

**FIGURE 2:**
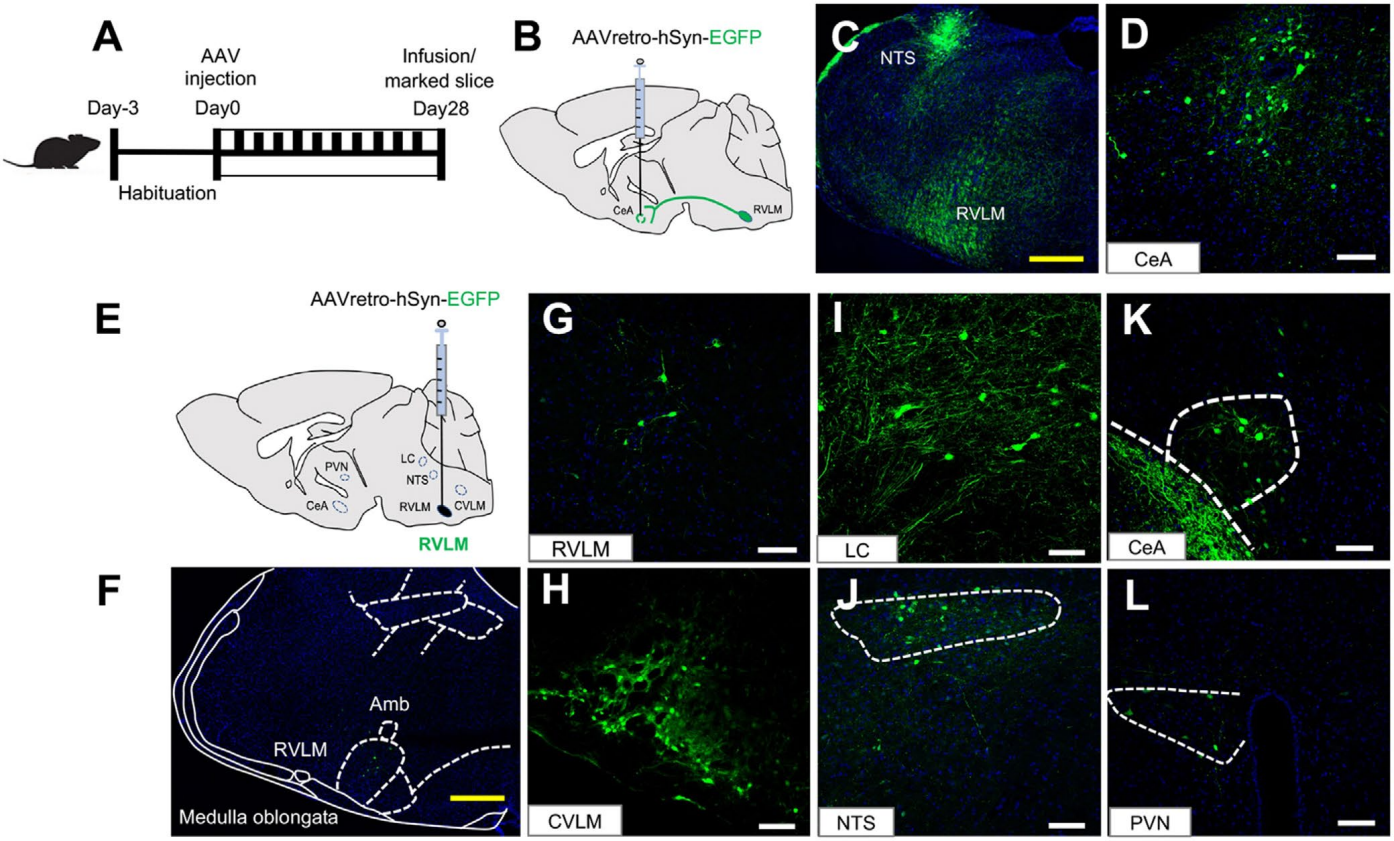
There is bidirectional projection in the RVLM and CeA. (A) Schematic illustrating the viral injection sites within the RVLM and CeA for retrograde neuronal tracing and marked brain slice acquisition. (B) AAVretro‐hSyn‐EGFP injection into the CeA were used to identify CeA‐projecting neurons in the RVLM. (C) Neurons labeled retrogradely in both the NTS and RVLM. Scale bar is 200 μm. (D) Representative photomicrographs confirming targeted viral delivery in the CeA. Scale bar is 100 μm. CeA‐projecting fibers are annotated at bregma −1.07 mm. (E) AAVretro‐hSyn‐EGFP injection into the RVLM were used to label RVLM‐projecting neurons in subcortical regions of the CeA. (F, G) Representative photomicrographs validating precise viral delivery into the RVLM. Yellow scale bar is 200 μm and white scale bar is 100 μm. RVLM‐associated fibers are indicated at bregma −6.6 mm. (H–L) Retrogradely labeled neurons observed in the CVLM, LC, NTS, CeA and PVN. Scale bars are 100 μm.

Given the pivotal featured of anxiety symptoms and BP variation under chronic light exposure [26, 33], we determined whether there is a correlation between the BP and anxiety‐like behaviors induced by chronic light. Especially, because of the direct or indirect effect of RVLM overexcitation on elevated BP [34], the neuronal excitation in the RVLM and CeA regions and the potential neural projection from the RVLM to CeA were further investigated (Figure 2). We examined the monosynaptic inputs of RVLM and CeA using a retrograde viral tracing strategy. After the mice adapted to environment for 3 days, the AAVretro‐hSyn‐EGFP vector was unilaterally injected into the RVLM and CeA, respectively. Four weeks after the viral injection, mice were perfused and the brain were carefully removed. Then the marked brain slices were obtained by screening the EGFP expression sections in the CeA and brainstem regions (Figure 2A). The RVLM retrograde tracing results showed that the EGFP expression was distributed in the caudal ventrolateral medulla (CVLM), locus coeruleus (LC), nucleus of solitary tract (NTS), CeA, and paraventricular nucleus (PVN) regions (Figure 2E–L). Importantly, the CeA retrograde tracing results have discovered that the EGFP expression in RVLM and NTS of the medulla oblongata were observed (Figure 2B–D). These labeled results have suggested that there is a bidirectional fiber connection between the RVLM and CeA, implying that the RVLM could transmit information to the midbrain CeA.

**FIGURE 3:**
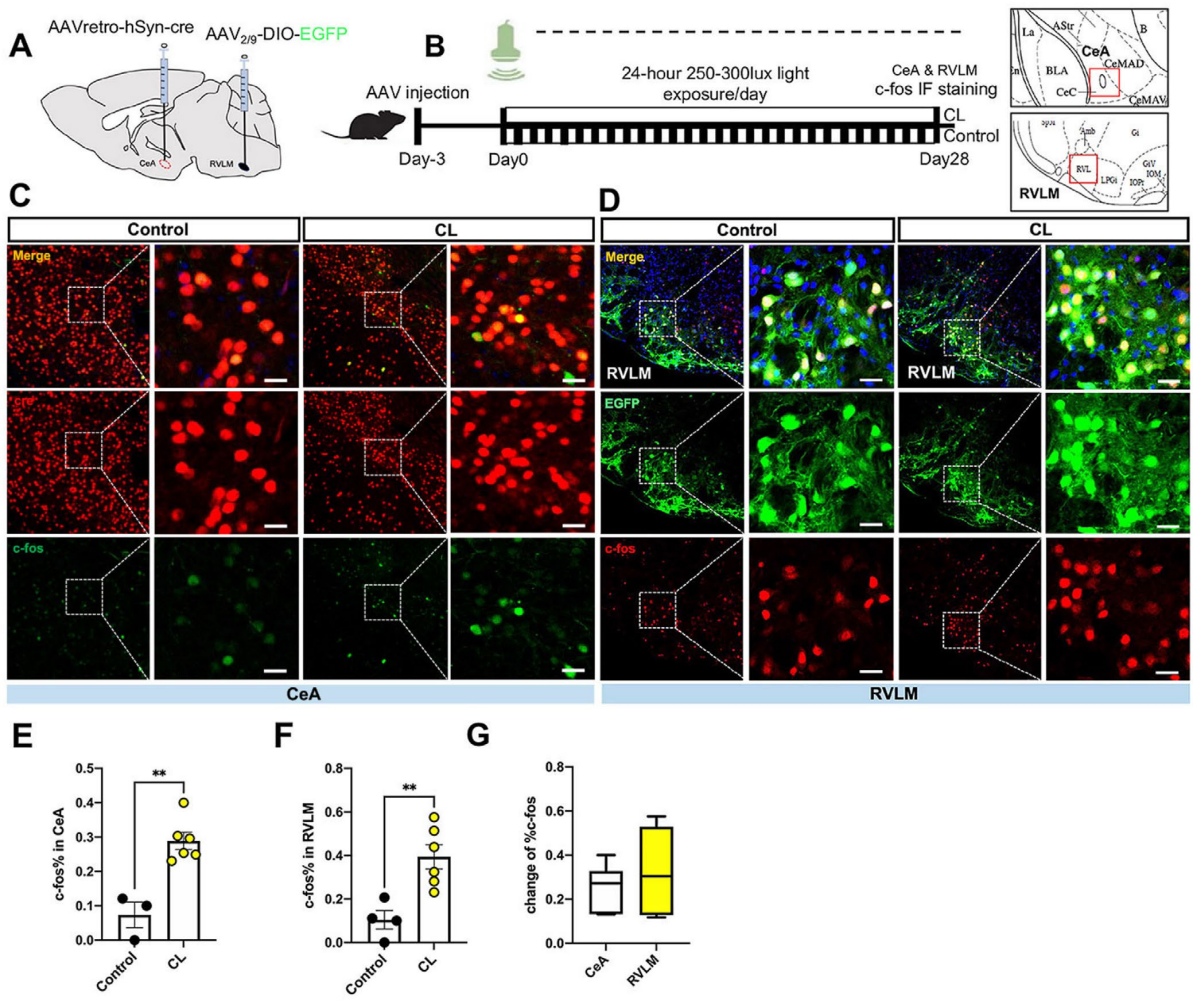
The c‐fos expression increased in the RVLM and CeA in CL mice. (A) Viral marking strategy: Schematic illustrating the dual‐virus retrograde tracing strategy used to specifically label RVLM neurons projecting to the CeA. AAVretro‐hSyn‐Cre was injected into the CeA and AAV_2/9_‐DIO‐EGFP was injected into the RVLM. (B) Timeline for light exposure and c‐fos staining in the CeA and RVLM. (C) The c‐fos expression was examined by immunofluorescence in the CeA (the left panel yellow scale bar is 100 μm in Control and CL groups, white dashed box scale bar is 20 μm). (D) The c‐fos expression was examined in the RVLM (the left panel yellow scale bar is 200 μm in Control and CL groups, white dashed box scale bar is 10 μm). (E) The c‐fos expression percentage of CeA neurons in the Control and CL mice. (F) The c‐fos expression percentage of RVLM neurons in the Control and CL mice. (G) The percentage difference value of c‐fos expression between the Control and CL mice in CeA and RVLM regions. Data are presented as mean ± SEM. Statistical significance was assessed by Student's unpaired *t*‐test (**p < 0.01). Sample sizes were as follows: For panel (E), *n* = 3 Control mice and *n* = 6 CL mice; for panels (F, G), *n* = 4 Control mice and *n* = 6 CL mice.

Further, the c‐fos expressions in the RVLM and CeA were measured. Based on the projection connection from RVLM to CeA, the dual‐virus retrograde tracing strategy was used to specifically label RVLM neurons projecting to the CeA. A retrograde AAV expressing Cre‐recombinase (AAVretro‐hSyn‐Cre) was injected into the CeA, while an AAV with a Cre‐dependent EGFP reporter (AAV‐DIO‐EGFP) was injected into the RVLM. This approach ensures that EGFP expression is restricted to RVLM→CeA projection neurons and the CeA neurons are marked by Cre recombinant protein. After viral injection for 4 weeks, the immunofluorescence staining was carried out to label Cre‐positive neurons (red) and c‐fos (green) in the CeA, and c‐fos positive cells (red) with EGFP colocalization in the RVLM. Finally, the c‐fos positive cells percentage was counted under the same field of view in the CeA and RVLM, respectively. We have found that the EGFP was strongly expressed in the RVLM neurons. The current results manifested that the c‐fos expressions significantly increased in the RVLM and CeA in CL mice relative to the Control mice (Figure 3A–F). Particularly, the increase of c‐fos expression was more pronounced in the RVLM than in the CeA after CL (Figure 3G), suggesting that CL leads to more enhanced neuronal excitation in the RVLM than in the CeA.

### 3.3 Chemogenetic Inhibitions of the RVLM and CeA Alleviated Anxiety‐Like Behaviors in CL Mice

**FIGURE 4:**
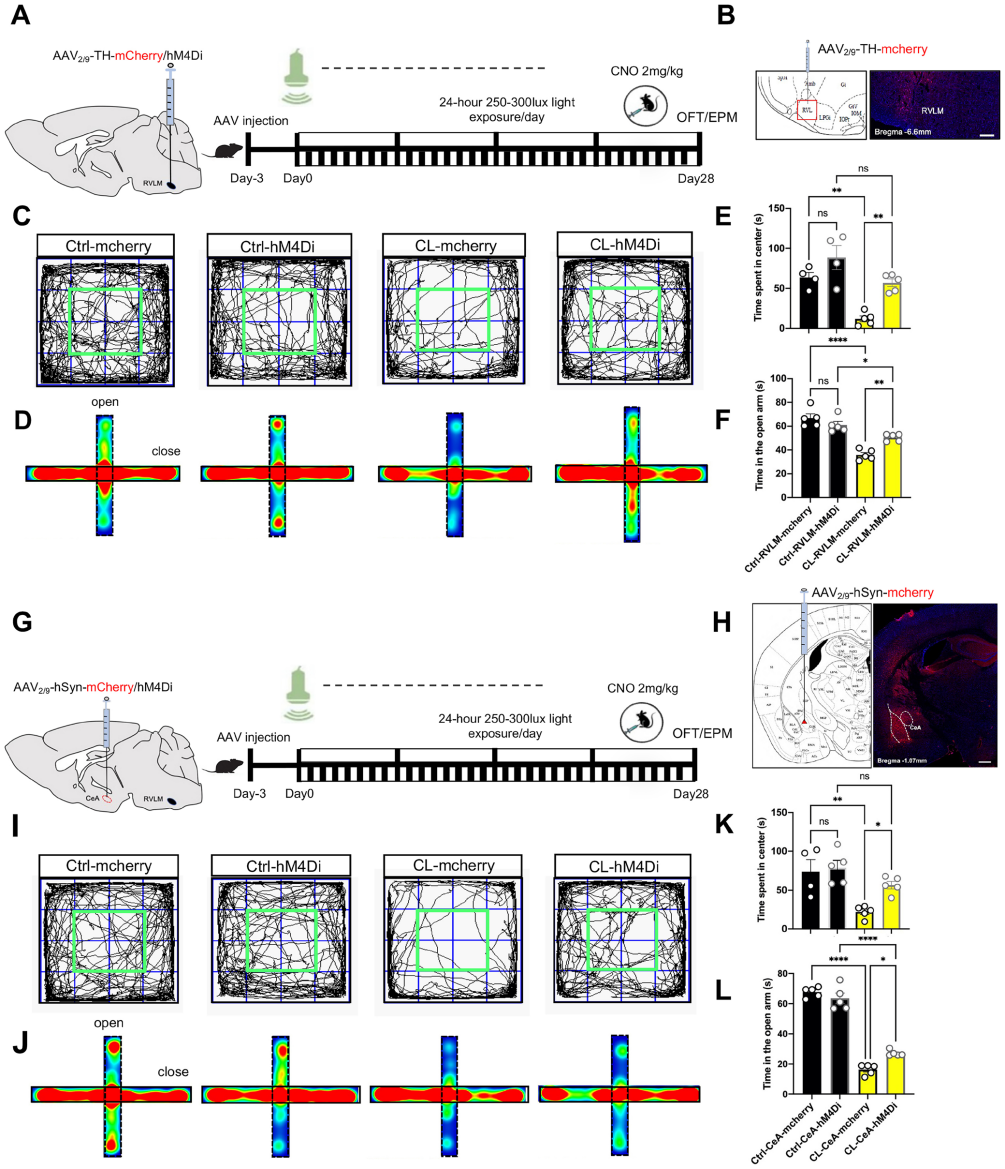
Chemogenetic inhibition of the RVLM and CeA notably alleviated anxiety‐like behaviors in CL mice. (A) Experimental process of TH positive neurons labeling and chemogenetic regulation in the RVLM. Mice were bilaterally injected with AAV_2/9_‐TH‐mcherry/hM4Di, and they were intraperitoneally injected with CNO (2 mg/kg) nearly 30 min before behavior tests. (B) The representative images of viral delivery sites within the RVLM (bregma −6.60 mm), scale bar is 200 μm. (C) The center movement trajectories comparison in the OFT after chemogenetic inhibition in the RVLM. (D) The corresponding movement heatmaps comparison in the EPM after chemogenetic inhibition in the RVLM. (E) Data summarizing the center zone duration in the OFT in the Ctrl‐mcherry, Ctrl‐hM4Di, CL‐mcherry and CL‐hM4Di groups. (F) Data summarizing time spent in the open arm of the EPM in the Ctrl‐mcherry, Ctrl‐hM4Di, CL‐mcherry and CL‐hM4Di groups. (G) Schematic of virus injection to label neurons and chemogenetic regulation in the CeA. Mice were bilaterally injected with AAV_2/9_‐hSyn‐mcherry/hM4Di, and they were intraperitoneally injected with CNO (2 mg/kg) nearly 30 min before behavior tests. (H) The representative images of the coronal slice showing the expression of mcherry in the CeA (bregma −1.07 mm), scale bar is 200 μm. (I) The center movement trajectories comparison in the OFT after chemogenetic inhibition in the CeA. (J) The movement heatmaps comparison in the EPM after chemogenetic inhibition in the CeA. (K) Data quantifying center zone duration of the OFT in the Ctrl‐mcherry, Ctrl‐hM4Di, CL‐mcherry and CL‐hM4Di mice. (L) Data quantifying open arm retention of the EPM in the Ctrl‐mcherry, Ctrl‐hM4Di, CL‐mcherry and CL‐hM4Di mice. Results are shown as mean ± SEM. Statistical significance indicated by *p < 0.05, **p < 0.01, ****p < 0.0001. Statistical analysis for panels (E), (F), (K), and (L) was performed using two‐way ANOVA. *n* = 5 for both mcherry and hM4Di‐expressing mice.

To examine the roles of inhibiting RVLM and CeA on regulating anxiety‐like behaviors induced by chronic light exposure, we next investigated the effects of chemogenetic inhibiting the RVLM (catecholaminergic‐CA neurons) [35] and CeA on anxiety‐like behaviors. To selectively manipulate the activity of catecholaminergic neurons in the RVLM, which are critically involved in the sympathetic response to stress, a tyrosine hydroxylase (TH) promotor AAV vector encoding the hM4Di receptor (a high‐affinity receptor for the pharmacologically inert ligand clozapine‐N‐oxide [CNO]) and the red fluorescent protein mcherry (AAV_2/9_‐TH‐hM4Di‐mcherry) was selectively expressed in CA neurons in the RVLM. Subsequently, the AAV_2/9_‐TH‐hM4Di‐mcherry vector was bilaterally injected into the RVLM and as a negative control, the AAV_2/9_‐TH‐mcherry vector also was bilaterally injected into the RVLM (Figure 4A,B). Three weeks after the viral injection, the neural activity of RVLM was inhibited following intraperitoneally CNO administration (Figure 4A). Behavioral analysis demonstrated an obvious increase in the center exploring duration of the OFT and the open arm retention of the EPM in CL‐RVLM‐hM4Di group relative to the CL‐RVLM‐mcherry group, and there were no significant differences in Ctrl‐RVLM‐hM4Di and Ctrl‐RVLM‐mcherry mice (Figure 4C–F), confirming that chemogenetic inhibiting of RVLM neuronal activity recovered CL mice's anxiety‐like responses.

Using the same strategy, chemogenetic inhibition of CeA resulted in the similar outcomes. A human synapsin (hSyn) promoter AAV vector encoding the hM4Di receptor and the red fluorescent protein mcherry (AAV_2/9_‐hSyn‐hM4Di‐mcherry) was selectively expressed in CeA neurons. Subsequently, the AAV_2/9_‐hSyn‐hM4Di‐mcherry/AAV_2/9_‐hSyn‐mcherry vectors were bilaterally injected into the CeA (Figure 4G,H). Three weeks after the viral injection, the neural activity of CeA was inhibited following intraperitoneally CNO administration (Figure 4G). The behavior results suggested that inhibiting the neuron activity in the CeA increased the exploring times in the center zone of OFT and the open arms of EPM in CL‐CeA‐hM4Di group comparation with the CL‐CeA‐mcherry group, but there had no differences among the Ctrl‐CeA‐hM4Di and Ctrl‐CeA‐mcherry mice (Figure 4I–L). Above all, the chemogenetic inhibition in the RVLM exhibited a greater effect (*p* < 0.01) than in the CeA (*p* < 0.05) in alleviating anxiety‐like behaviors (Figure 4E,F,K,L). These data indicated that chemogenetic inhibition of the RVLM and CeA was sufficient to recover anxiety disorder in CL mice. As anticipated, CL elicited anxiety‐like phenotypes that were partially attenuated by chemogenetic inhibition of the RVLM and CeA in mice.

### 3.4 Optogenetic Manipulation of the RVLM→CeA Pathway Partially Alleviated Anxiety‐Like Behaviors in the CL Mice

**FIGURE 5:**
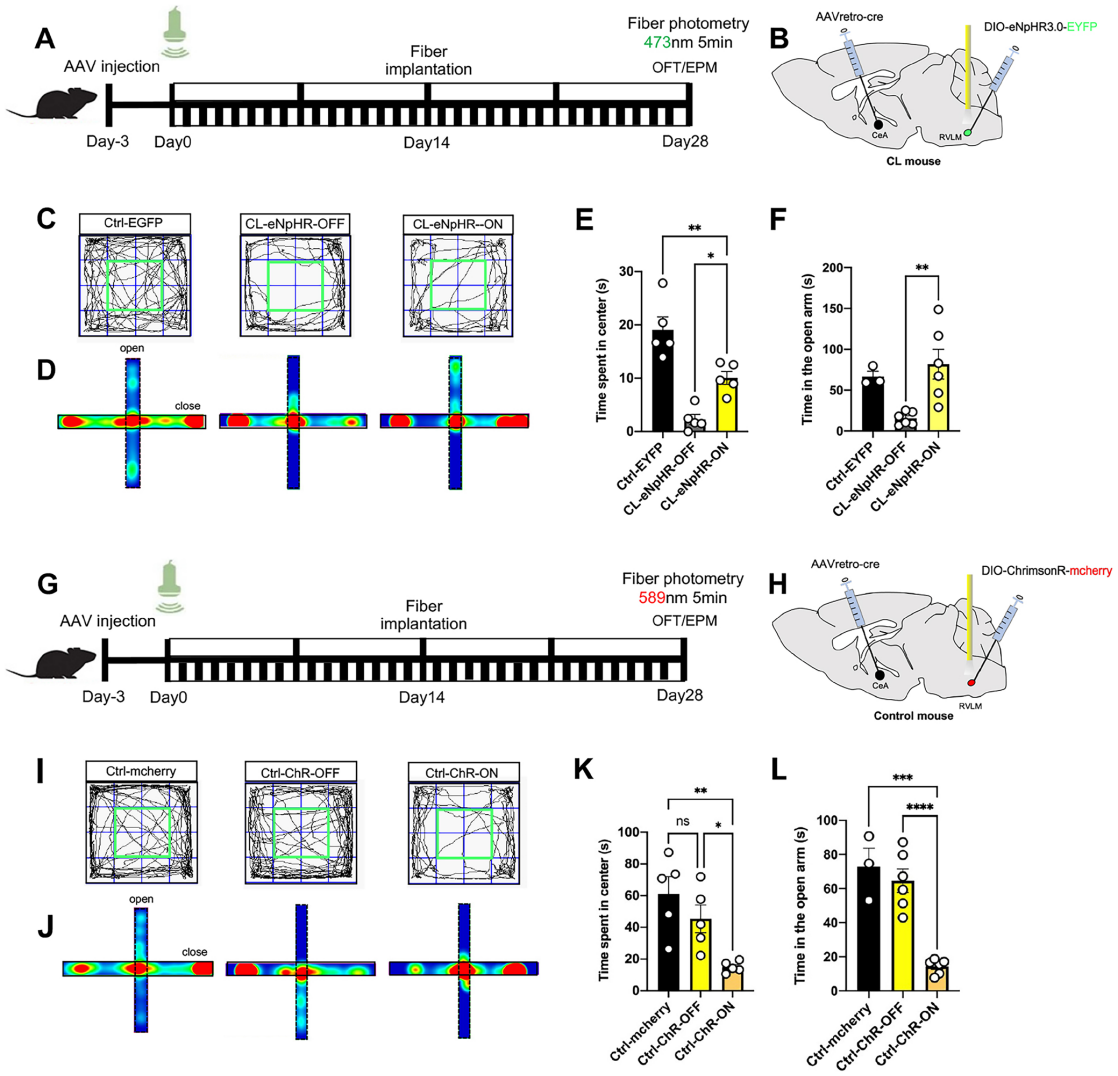
Optogenetic inhibition of the RVLM→CeA pathway significantly recovered anxiety‐like behaviors in CL mice. (A) Schematic of optogenetic virus injection, fiber implantation and behavior tests during optogenetic inhibition in control and CL mice. (B) Neurocircuit marking strategy as follows: inhibitory optogenetic virus injection (DIO‐eNpHR3.0‐EYFP) targeting CeA‐projecting neurons in the RVLM. (C) The movement trajectories of OFT in the Ctrl‐EYFP, CL‐eNpHR‐OFF and CL‐eNpHR‐ON groups during optogenetic inhibition after laser‐on. (D) Representative heatmaps of EPM in the Ctrl‐EYFP, CL‐eNpHR‐OFF and CL‐eNpHR‐ON groups during optogenetic inhibition after laser‐on. (E) Data summarizing center zone duration of the OFT in the Ctrl‐EYFP, CL‐eNpHR‐OFF and CL‐eNpHR‐ON groups after laser‐on. (F) Summarized data for time in the open arms of EPM in the Ctrl‐EYFP, CL‐eNpHR‐OFF and CL‐eNpHR‐ON groups after laser‐on. (G) Schematic of optogenetic virus injection, fiber implantation and behavior tests during optogenetic activation in control and CL mice. (H) Excitatory optogenetic virus injection (DIO‐ChrimsonR‐mcherry) targeting CeA‐projecting neurons in the RVLM. (I) The movement trajectories of OFT in the Ctrl‐mcherry, Ctrl‐ChR‐OFF and Ctrl‐ChR‐ON groups during optogenetic activation after laser‐on. (J) Representative heatmaps of EPM in the Ctrl‐mcherry, Ctrl‐ChR‐OFF and Ctrl‐ChR‐ON groups during optogenetic activation after laser‐on. (K) Data summarizing center zone duration of the OFT in the Ctrl‐mcherry, CL‐ChR‐OFF and CL‐ChR‐ON groups after laser‐on. (L) Summarized data for time in the open arms of EPM in the Ctrl‐mcherry, CL‐ChR‐OFF and CL‐ChR‐ON groups after laser‐on. Data are expressed as mean ± SEM. Significance is indicated as follows: *p < 0.05; **p < 0.01; ***p < 0.001; ****p < 0.0001. One‐way ANOVA was used for analysis of panels (E), (F), (K), and (L). For (E), *n* = 5 Ctrl‐EYFP and CL‐eNpHR mice. For (F), *n* = 3 Ctrl‐EYFP, *n* = 6 CL‐eNpHR mice. For (K), *n* = 5 Ctrl‐mcherry and Ctrl‐ChR mice. For (L), *n* = 3 Ctrl‐mcherry, *n* = 6 Ctrl‐ChR mice.

Above results suggest that chronic light exposure was transmitted to activate the RVLM and CeA regions, leading to maladaptive anxiety‐like behaviors. Given the importance of the impact of neuronal excitation in RVLM on anxiety‐like behaviors, further, we examined whether the pathway from the RVLM to CeA was necessary to lead to anxiety‐like behaviors evoked by chronic light exposure. First, the viruses of AAVrerto‐hSyn‐cre and a Cre‐dependent AAV vector encoding a genetically engineered eNpHR3.0/ChrimsonR genes and EGFP/mcherry proteins (rAAV‐DIO‐eNpHR3.0‐EGFP/rAAV‐DIO‐ChrimsonR‐mcherry) were respectively transfected into the CeA and RVLM in the CL and Control mice (Figure 5A,B,G,H). Two weeks after the viral injection, the optical fiber implantation was carried out. After completing a light exposure regimen and 4 weeks' viral injection simultaneously mice were subjected to optogenetic inhibition of the pathway from the RVLM to CeA in CL mice. Then, their behaviors were examined during five min laser on or off. It was found that CL‐eNpHR‐ON mice exhibited a significant increase in center exploring duration of the OFT and extended open arm retention time of the EPM comparation with CL‐eNpHR‐OFF mice upon optical inhibition, suggesting optogenetic inhibition of the RVLM→CeA pathway partially recovered the CL mice's anxiety‐like behaviors and produced a statistically significant and substantial reduction, but not completely reversed the anxiety‐like phenotype (Figure 5C–F). Similarly, after optical fiber implantation and 4 weeks' viral expression, the optogenetic activation was manipulated from the RVLM to CeA in Control mice (Figure 5G,H). A key observation was that optogenetic stimulation of the RVLM–CeA pathway in Control mice elicited obvious anxiogenic effects, as evidenced by behavioral performance in both the OFT and EPM. Specifically, upon light activation, stimulated Control mice exhibited evident reductions in the center zone occupancy of OFT and the open arm time of EPM in the Ctrl‐ChR2‐ON mice relative to the Ctrl‐ChR2‐OFF mice (Figure 5I–L). These cumulative results suggested that optogenetic inhibition of the RVLM input to CeA alleviated anxiety‐like behaviors in CL model mice, whereas its optogenetic excitation acutely evoked anxiety‐like behaviors in the normal mice, indicating that the augmented excitatory drive from the RVLM to CeA was necessary to regulate the anxiety‐like behaviors under the chronic light exposure.

## 4 Discussion

This study demonstrates that chronic light (CL) exposure induces robust anxiety‐like behaviors in mice and identifies a previously underappreciated neural mechanism involving the RVLM→CeA projection. Under CL conditions, excitatory neuronal activity was markedly elevated in both the RVLM and CeA regions, with greater hyperexcitability observed in the RVLM. Importantly, chemogenetic and optogenetic manipulation of the RVLM→CeA pathway significantly alleviated anxiety‐like behaviors, underscoring the essential role of this circuit in mediating emotional response to aberrant light exposure.

The CeA has long been recognized as a critical hub for regulating stress‐related anxiety behaviors [25, 36, 37]. Previous studies have suggested that projections from the CeA to the RVLM contribute to the sympathetic activation observed during fear and psychological stress [38, 39, 40]. The CeA receives upstream inputs from the lateral hypothalamus (LH), bed nucleus of the stria terminalis (BNST), and periaqueductal gray (PAG), thereby integrating limbic, hypothalamic, and brainstem information to generate coordinated emotional and physiological responses [30, 41, 42, 43, 44, 45]. Our anatomical tracing and functional data complement these findings by identifying a reciprocal excitatory projection from the RVLM to the CeA, providing a direct mechanistic link between autonomic and emotional processing under environmental stress. Abnormal sympathetic excitation and hypertension have been shown to cause vascular remodeling and cerebral hypoperfusion, impairing prefrontal inhibitory control over the amygdala and exacerbating anxiety [32]. Given the pivotal role of the RVLM under light exposure [26, 33], our c‐fos analyses revealed stronger neuronal activation in the RVLM than in the CeA in the CL‐exposed mice, indicating that the RVLM may act as the primary driver of limbic hyperactivity under chronic light stress. Moreover, chemogenetic inhibition of RVLM neurons markedly reduced anxiety‐like behaviors, confirming the pivotal contribution of this brainstem nucleus to affective dysregulation. In addition to central effects, CL exposure increased systolic and diastolic blood pressure as well as plasma norepinephrine and epinephrine levels, indicating elevated sympathetic output. These systemic changes suggest that heightened peripheral catecholamine signaling may convey stress‐related information back to the central nervous system, further amplifying RVLM→CeA excitability. Together, these findings support the hypothesis that abnormal sympathetic excitation originating in the RVLM can influence limbic circuits such as the CeA to drive anxiety‐like phenotypes. These extends prior evidence [21] by functionally implicating this brainstem‐amygdala circuit in environmental light‐induced emotional dysregulation.

Excessive light exposure has been reported to suppress melatonin secretion and impair cognition and mood in rodents [46, 47, 48, 49, 50]. In line with these findings, our results showed elevated plasma glucocorticoids and corticosterone levels accompanied by a trend towards reduced melatonin in CL‐exposed mice. Moreover, chronic light exposure markedly decreased spontaneous activity and disrupted circadian wheel‐running patterns (Figure S2), suggesting that increased stress susceptibility under CL may be associated with circadian rhythm disturbances and altered neuroendocrine homeostasis.

Beyond its classical role in maintaining blood pressure and sympathetic tone, the RVLM may serve as a convergent node where environmental stressors, such as chronic light exposure, initiate cascades of neuro‐autonomic and affective alterations [51, 52, 53]. Our findings highlight that light‐induced RVLM hyperactivity is not an isolated physiological response but rather an integrated process involving direct communication with limbic emotional centers. Under chronic light conditions, increased excitatory output from the RVLM to the CeA may be reciprocally reinforced by CeA‐to‐RVLM projections, forming a positive feedback loop that sustains sympathetic hyperactivation and anxiety‐like behaviors. This reciprocal facilitation provides a plausible mechanistic explanation for the well‐documented comorbidity between hypertension and mood disorders, emphasizing that autonomic dysregulation contributes to the pathogenesis of anxiety.

**FIGURE 6:**
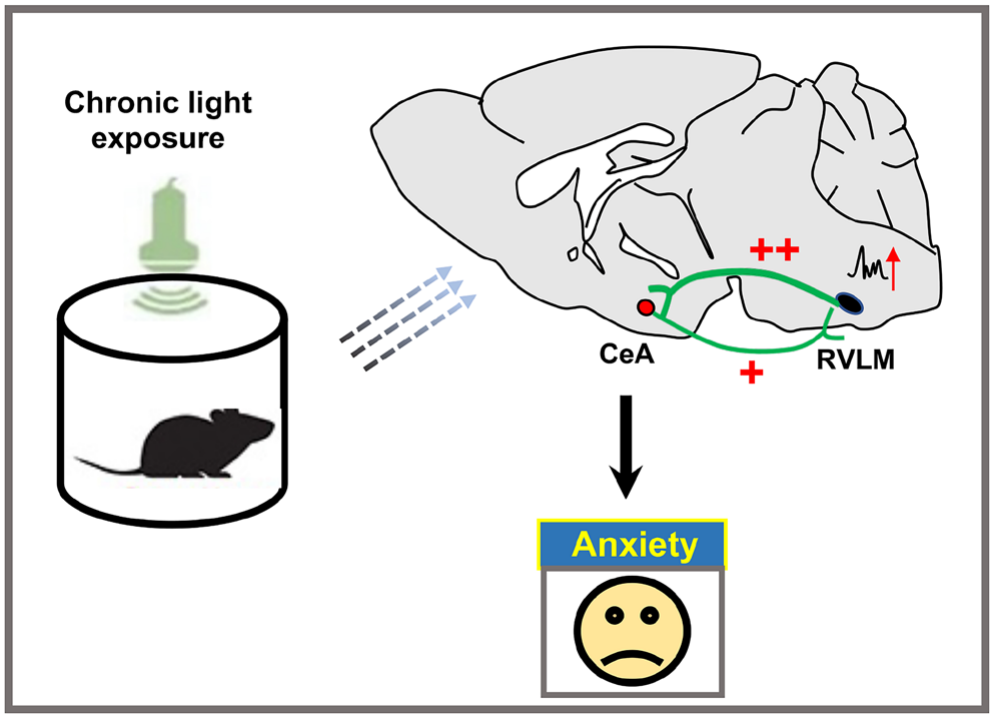
The augmented excitatory drive from the RVLM to CeA regulates anxiety‐like behaviors under chronic light exposure.

In summary, our findings propose a model in which chronic light exposure acts as an environmental stressor that enhances RVLM excitability, elevates sympathetic drive, and modulates CeA activity through direct projections, ultimately resulting in anxiety‐like behaviors. These identification of this RVLM→CeA axis provides a novel neurobiological framework linking environmental perturbations to affective dysfunction (Figure 6). Future studies should further investigate the therapeutic potential of targeting this brainstem‐amygdala pathway for mood disorders associated with autonomic imbalance and environmental stress.

### 4.1 Limitation

While our study primarily focused on the sympathetic arm of the RVLM, the role of parasympathetic activity, particularly vagal tone, warrants further attention. The CeA receives viscerosensory information through projections from brainstem nuclei, and impaired vagal signaling may alter CeA excitability and emotional reactivity [54, 55]. We investigated RVLM activity within the brainstem under CL, establishing the necessity of RVLM input to the CeA for CL exposure‐induced anxiety‐like behaviors. It did not specifically address the effects of physiological or pathological disconnection of sympathetic outflow on these behaviors under light exposure conditions and did not directly prove that the high blood pressure and behavioral effects share a single causal mechanism. The direct functional evidence for the critical role of the RVLM→CeA projection in light‐induced anxiety, and it is also important to note the reciprocal CeA→RVLM pathway. Therefore, the RVLM→CeA bidirectional feedback loop, while consistent with known anatomy and our functional data, remains a hypothesis that requires future validation. Additionally, this study was conducted exclusively in male mice. While this approach controlled for potential confounding effects of the estrous cycle, it limits the generalizability of our findings. Future studies are necessary to determine whether the RVLM→CeA pathway plays a similar, enhanced, or diminished role in mediating light‐induced anxiety in female subjects. Despite these limitations, our data extend our current understanding of the mechanisms underlying CL exposure‐induced anxiety, elevated blood pressure, and heightened sympathetic activity. Collectively, this study establishes a conceptual framework for elucidating the comorbidity of hypertension and anxiety, notably among occupational groups exposed to excessive artificial light.

## Author Contributions

Jia‐Ni Jing, Xing Tan, and Wei‐Zhong Wang designed the study. Jia‐Ni Jing completed the retrograde tracing, behavior tests, immunofluorescent staining, ELISA, optogenetic and chemogenetic experiments, and blood pressure recording. Chao Yuan did confocal image analysis. Jia‐Ni Jing, Xing Tan, Hao Fan, and Chou Xu analyzed the data. Jia‐Ni Jing established the light‐exposed model mice and wrote the discussion and comments on the manuscript. Wei‐Zhong Wang supervised the project.

## Funding

This work was supported by National Natural Science Foundation of China, 32100954, 82270467.

## Ethics Statement

The study complied with the NIH Guide for the Care and Use of Laboratory Animals and was approved by the Institutional Animal Care and Use Committee (IACUC) of the Military Medical Research Institute (Approval ID: IACUC‐JSRZ‐23‐0201).

## Conflicts of Interest

The authors declare no conflicts of interest.

## Supporting information


**Figure S1:** The decreased 24 h sucrose consumption in CL mice. (A) Schedule of chronic light exposure and sucrose preference test (SPT). (B, C) Summarized data for 24 h sucrose consumption (%) at different time points of light exposure (1 W–4 W). The data are expressed as the mean ± SEM. CL vs. Control ***p < 0.001; ****p < 0.0001 in the 1 W, 2 W, 3 W and 4 W respectively. Repeated Measurement ANOVA analysis for (B, C). *n* = 5 Control and CL mice.
**Figure S2:** Chronic light exposure led to a reduced spontaneous activity and increased corticosterone and glucocorticoid in CL mice. (A) Schedule of chronic light exposure and wheel running test. (B) The locomotor activity recording in Control mice for 15 days (7:00 light on and 19:00 light off). (C) The locomotor activity recording in CL mice for 15 days (7:00 light on and 19:00 light off). (D) The counts of every hour in the Control and CL mice. (E) Plasma concentrations of melatonin in control and CL mice. (F) Plasma concentrations of corticosterone in Control and CL mice. (G) Plasma concentrations of glucocorticoid in Control and CL mice. The data are expressed as the mean ± SEM. **p < 0.01; ***p < 0.001; ****p < 0.0001. Unpaired *t*‐test analysis for (D–G). For (D, E) *n* = 6 Control mice, *n* = 7 CL mice. For (F, G) *n* = 6 Control and CL mice.

## Data Availability

All data that support the findings of this study are available on request from the corresponding author. The data are not publicly available due to privacy or ethical restrictions.
