# Supplementary material for: The Rostral Ventrolateral Medulla Input to Central Amygdala Regulates Anxiety‐Like Behaviors in Mice for Chronic Light Exposure
Source: CNS Neurosci Ther. 2026 Feb 5;32(2):e70775. doi: 10.1002/cns.70775 (PMC12877313; doi:10.1002/cns.70775)

Supplementary Figure 1

**The decreased 24h sucrose consumption in CL mice**. (A) Schedule of chronic light exposure and sucrose preference test (SPT). (B-C) Summarized data for 24 h sucrose consumption (%) at different time points of light exposure (1W-4W). The data are expressed as the mean ± SEM. CL *vs.* Control ****p*<0.001; *****p*<0.0001 in the 1W, 2W, 3W and 4W respectively. Repeated Measurement ANOVA analysis for (B-C). n=5 Control and CL mice.

Supplementary Figure 2

**Chronic light exposure led to a reduced spontaneous activity and increased corticosterone and glucocorticoid in CL mice**. (A) Schedule of chronic light exposure and wheel running test. (B) The locomotor activity recording in Control mice for 15days (7:00 light on and 19:00 light off). (C) The locomotor activity recording in CL mice for 15days (7:00 light on and 19:00 light off). (D) The counts of every hour in the Control and CL mice. (E) Plasma concentrations of melatonin in control and CL mice. (F) Plasma concentrations of corticosterone in Control and CL mice. (G) Plasma concentrations of glucocorticoid in in Control and CL mice. The data are expressed as the mean ± SEM. ***p<*0.01; ****p<*0.001; *****p*<0.0001. Unpaired *t-test* analysis for (D-G). For (D, E) n=6 Control mice, n=7 CL mice. For (F, G) n=6 Control and CL mice.


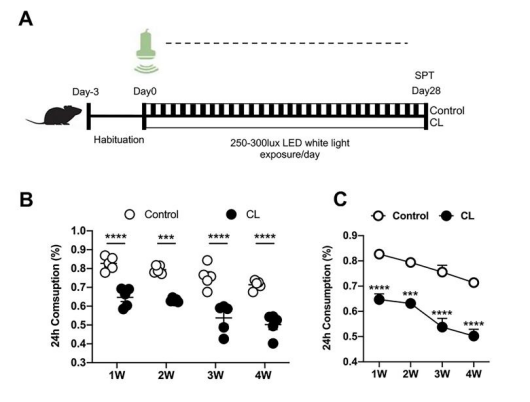
**Supplementary Figure 1**

**Supplementary Figure 2**


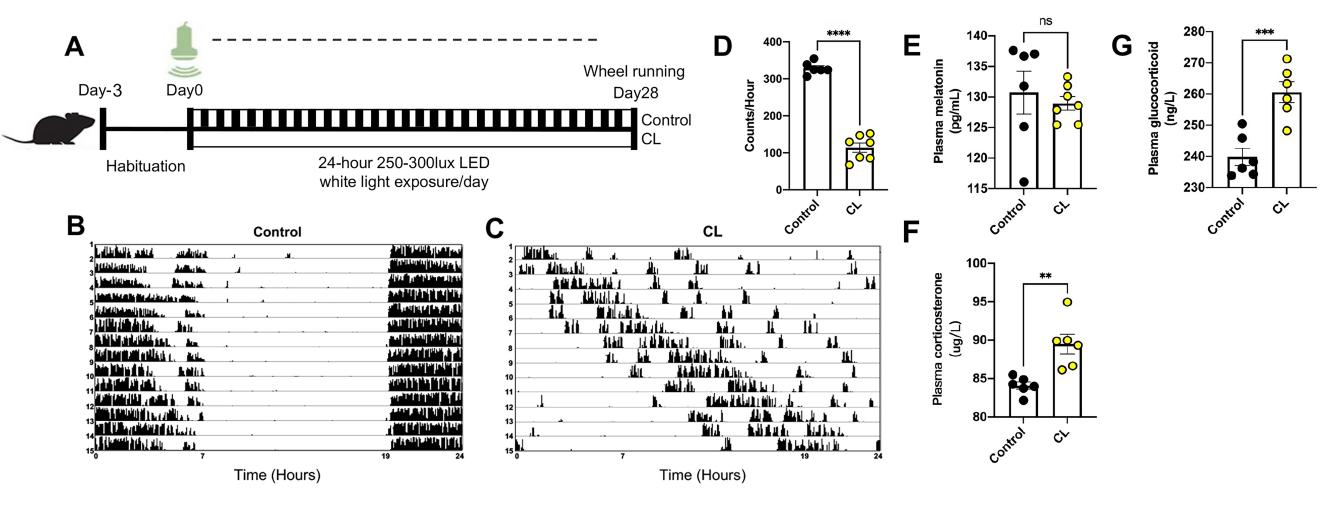

Supplement: Supplementary file 1 — Figure S1: The decreased 24 h sucrose consumption in CL mice. (A) Schedule of chronic light exposure and sucrose preference test (SPT). (B, C) Summarized data for 24 h sucrose consumption (%) at different time points of light exposure (1 W–4 W). The data are expressed as the mean ± SEM. CL vs. Control ***p < 0.001; ****p < 0.0001 in the 1 W, 2 W, 3 W and 4 W respectively. Repeated Measurement ANOVA analysis for (B, C). n = 5 Control and CL mice. Figure S2: Chronic light exposure led to a reduced spontaneous activity and increased corticosterone and glucocorticoid in CL mice. (A) Schedule of chronic light exposure and wheel running test. (B) The locomotor activity recording in Control mice for 15 days (7:00 light on and 19:00 light off). (C) The locomotor activity recording in CL mice for 15 days (7:00 light on and 19:00 light off). (D) The counts of every hour in the Control and CL mice. (E) Plasma concentrations of melatonin in control and CL mice. (F) Plasma concentrations of corticosterone in Control and CL mice. (G) Plasma concentrations of glucocorticoid in Control and CL mice. The data are expressed as the mean ± SEM. **p < 0.01; ***p < 0.001; ****p < 0.0001. Unpaired t‐test analysis for (D–G). For (D, E) n = 6 Control mice, n = 7 CL mice. For (F, G) n = 6 Control and CL mice. [file CNS-32-e70775-s001.docx]
